# Cardiac Multimodality Imaging Assessment of Dystrophic Myocardial Calcification in a Human Immunodeficiency Virus-Infected Patient With Dilated Cardiomyopathy

**DOI:** 10.7759/cureus.18707

**Published:** 2021-10-12

**Authors:** Diego Xavier Chango Azanza, Beatriz Fernández, Zuilma Vásquez Ortiz, Mónica Chapa, Sandra Rosales Uvera

**Affiliations:** 1 Department of Cardiovascular Imaging, National Institute of Medical Sciences and Nutrition Salvador Zubiran, Mexico City, MEX; 2 Department of Echocardiography, National Institute of Medical Sciences and Nutrition Salvador Zubiran, Mexico City, MEX

**Keywords:** dystrophic myocardial calcification, cardiac multimodality imaging, hiv-infection

## Abstract

Dystrophic myocardial calcification represents the sequelae of local tissue damage and cellular necrosis. We present the case of a 72-year-old man who presented with exertional chest pain. He had a medical history of human immunodeficiency virus (HIV) infection and chronic dilated cardiomyopathy with severe left ventricular (LV) systolic dysfunction and wall motion abnormalities at the inferior and lateral LV walls. A cardiac magnetic resonance (CMR) examination from 16 years ago showed a subendocardial late gadolinium enhancement (LGE) distribution consistent with prior myocardial infarction (MI). Recently, a pharmacological stress myocardial perfusion imaging by CMR had been positive for myocardial ischemia in the left descending coronary artery (LAD) territory. A cardiac CT angiography (CCTA) showed non-significant LAD obstruction <50% consistent with microvascular ischemia and the presence of dystrophic myocardial calcification as an unusual progression of a prior MI. Conservative approach and optimal medical therapy were employed in our patient, and there was no symptom progression during the two-month follow-up period.

## Introduction

Patients with human immunodeficiency virus (HIV) infection can have a variety of cardiac and vascular manifestations. When cardiomyopathy is present, the precise pathophysiology is often unclear but is probably multifactorial. Some patients with asymptomatic or overt left ventricular (LV) dysfunction have known or clear etiologies such as coronary artery disease, cocaine use, alcoholic heart disease, drug toxicity, or myocarditis due to an opportunistic infection such as toxoplasmosis or cryptococcosis [1-3].

The calcification of the myocardium is an unusual finding that can arise from several different causes and indicates an underlying pathology associated with morbidity and mortality [4]. Pathologic calcification in any tissue represents an abnormal accumulation of calcium salts, and in the myocardium, two basic forms are recognized: dystrophic and metastatic [5]. Dystrophic is more prevalent than metastatic calcification, and the most common etiology is previous myocardial infarction (MI) leading to myocyte necrosis [5,6].

A multimodality cardiac imaging approach would be useful in this context for evaluating the diagnosis and clarifying the myocardial involvement. Echocardiography is the first-line cardiac imaging modality to assess the myocardium, pericardium, valves, LV systolic and diastolic dysfunction, and segmentary myocardial thickening and contractility. Cardiac magnetic resonance (CMR) exam is the gold standard technique to evaluate LV volumes and ejection fraction, and it also enables the study of etiology in the setting of cardiomyopathy by late gadolinium enhancement (LGE) pattern. Moreover, cardiac CT angiography (CCTA) is an attractive technique to complement the evaluation of epicardial coronary artery disease, the characterization of plaque burden, and to morphologically determine LV involvement thanks to its high spatial resolution and the unique capability to characterize calcifications in several structures.

## Case presentation

**Figure 1:**
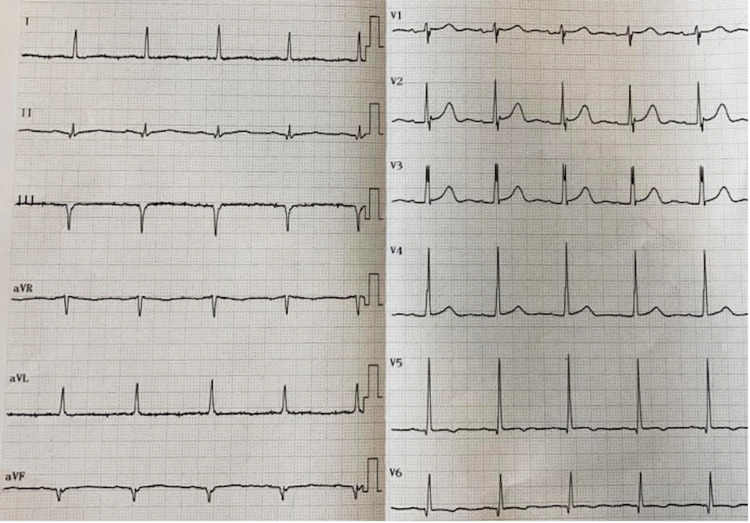
12-lead electrocardiogram of the patient

A 72-year-old male presented with chest pain on exertion for one year. It was stated that it disappears in a few minutes after effort cessation. He had a Class II New York Heart Association (NYHA) functional status with only a slight limitation of physical activity but without symptoms at rest. He had a medical history of HIV infection 30 years ago under antiretroviral therapy (lopinavir/ritonavir), dyslipidemia treated with atorvastatin 20 mg per day, deep vein thrombosis on oral anticoagulation, and chronic dilated cardiomyopathy. He had had undetectable viral load test results for at least six months. His electrocardiogram showed sinus rhythm, a heart rate of 62 beats per minute with the presence of pathological "Q" waves, and a lack of "R" wave voltage in the inferior leads (DII, DIII, and aVF), and ST-T segment abnormalities at the inferolateral precordial leads (v5-v6) (Figure 1).

**Figure 2:**
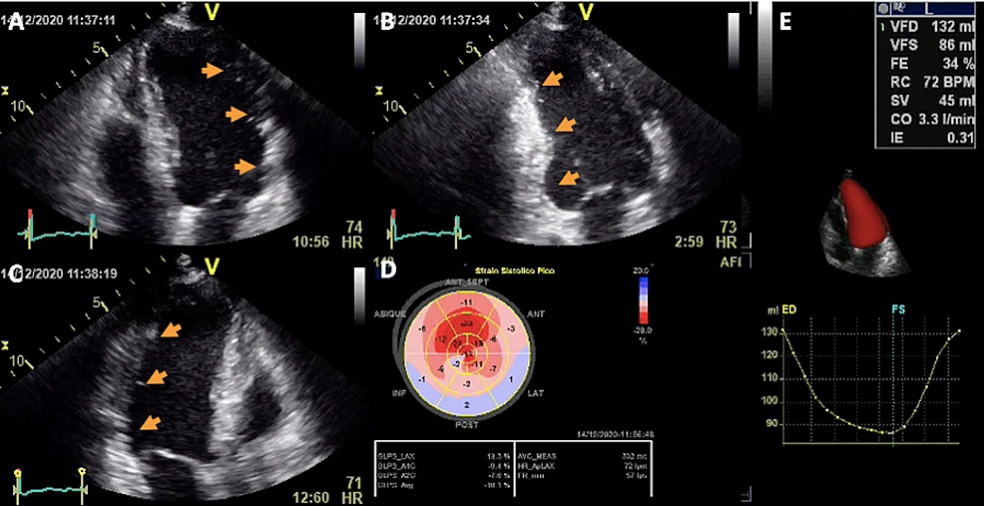
Transthoracic echocardiography findings of the patient Two-dimensional left ventricular apical views at the A: four-chamber view, B: two-chamber view, and C: three-chamber view. D: bullseye map of left ventricular global longitudinal strain. E: three-dimensional echocardiography evaluation of left ventricular volumes and ejection fraction

The transthoracic echocardiography denoted severe LV dysfunction, and an extensive akinesis and echo-hyperrefringency from basal to apex at the inferior, inferolateral, and lateral LV walls. Three-dimensional echocardiography allowed us to estimate an LV end-diastolic volume of 132 ml, LV end-systolic volume of 86 ml, and LV ejection fraction of 34%. The global longitudinal strain was calculated as -13.3%, which was compatible with abnormal values in the context of a chronic dilated cardiomyopathy (Figure 2, Video 1).

**Video 1:** Transthoracic echocardiography findings of the patient Two-dimensional apical left ventricular assessment images at the four-chamber view (upper left), two-chamber view (upper right), three-chamber view (bottom left), and bullseye map of left ventricular global longitudinal strain (bottom right)

**Figure 3:**
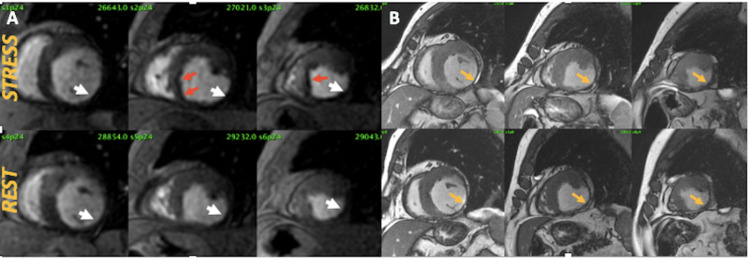
Pharmacological stress myocardial perfusion imaging by CMR using dipyridamole A: left ventricular short-axis views from base to apical segments in stress (upper left) and rest (bottom left) showing permanent perfusion defect at the inferior, inferolateral, and lateral left ventricle from base to apex (white arrows) and reversible perfusion defect at the mid-inferoseptal, anteroseptal, and septal apical segments (red arrows). B: cine steady-state free precession short-axis images during systole in stress (upper right) and rest (bottom right) denoting a very thinned and akinetic motion at the inferior, inferolateral, and lateral left ventricle from base to apex (orange arrows) CMR: cardiac magnetic resonance

CMR pharmacological stress myocardial perfusion imaging was chosen to confirm the presence and for quantifying the amount of myocardial ischemia related to the exertional chest pain in our patient. Dipyridamole was used at doses of 0.84 mg/kg infused in four minutes. Neither any cardiac symptoms such as chest pain or dyspnea nor other drug infusion-related complications were present during the test. However, imaging analysis denoted a persistent perfusion defect in thinned and akinetic LV walls, and there was a reversible perfusion defect in mid-inferoseptal, anteroseptal, and septal apical LV walls indicating myocardial ischemia in left descending coronary artery (LAD) territory (Figure 3).

**Figure 4:**
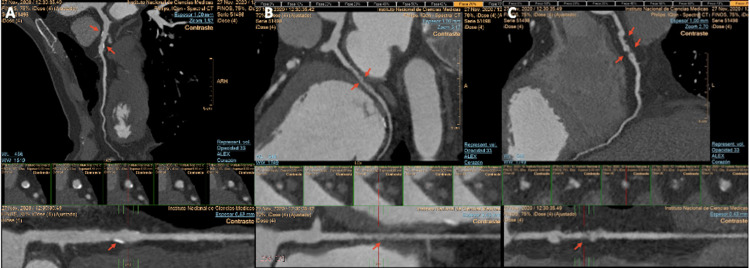
Coronary CT angiography findings of the patient A: left descending coronary artery; B: circumflex coronary artery; and C: right coronary artery with a diffuse multi-vessel disease with calcified and non-calcified plaque burden CT: computed tomography

In the context of myocardial ischemia on the LAD coronary artery territory, a CCTA was preferred with a non-invasive approach to determine atherosclerotic coronary artery disease and myocardial involvement in our patient. A diffuse non-significant obstructive LAD atherosclerotic coronary disease with calcified plaques at the proximal and mid-segments (25-49% obstruction) was observed. Therefore, myocardial ischemia was found to be present in a <50% coronary artery obstruction, suggesting a Coronary Vasomotor Disorders International Study Group (COVADIS) type 2 microvascular ischemia. On the other hand, the circumflex coronary artery had a diffuse non-calcified disease at the mid-segment causing a non-significant obstruction (25-49%), and the right coronary artery (RCA) showed diffuse involvement with non-calcified plaques at the proximal and mid-segments. There was a significant obstruction at the level of the mid-RCA (50-69% coronary stenosis) (Figure 4). Direct functional coronary artery assessment of fractional flow reserve (FFR) was not available in our case, and neither was non-invasive FFR-CT.

**Figure 5:**
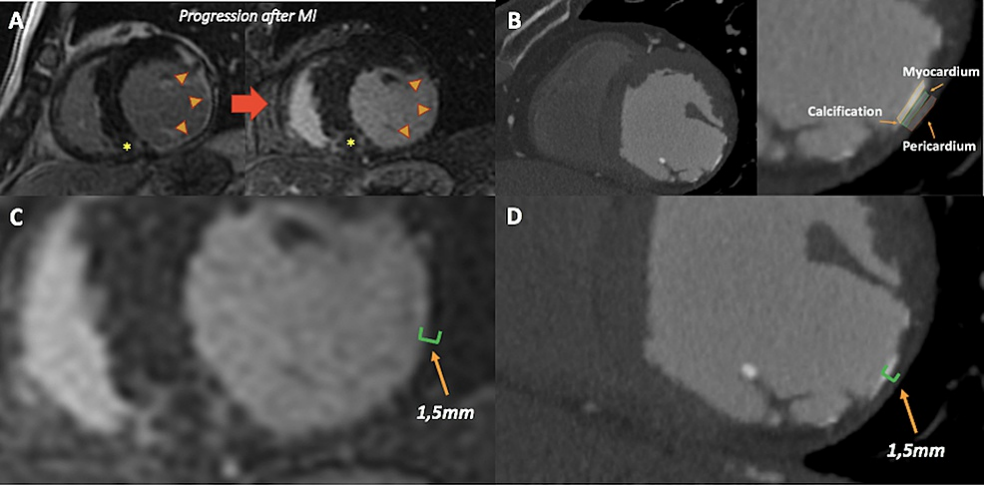
CMR and CT findings A: cardiac magnetic short-axis images showing the progression over 16 years, of the evolution of an extensive myocardial infarction with subendocardial LGE distribution (orange arrows), and a non-ischemic patchy intramyocardial LGE at the basal inferoseptal level (yellow asterisks). B: CCTA short-axis images at the same level confirming the dystrophic calcification of a part of the myocardium at the subendocardial level. C and D: comparative CMR and CCTA assessment of myocardial involvement in short-axis left ventricular views MI: myocardial infarction; LGE: late gadolinium enhancement; CCTA: cardiac computed tomography angiography; CMR: cardiac magnetic resonance

Additionally, we were able to accurately characterize the LV involvement by CCTA, and as an unusual finding, the calcification of the myocardium was present at the levels of LV thinned walls. A CMR examination from 16 years ago had already shown the presence of severe LV systolic dysfunction and dilatation with the extensive compromise of LV walls. Furthermore, LGE was present in a subendocardial distribution with a transmural infarction of 70% of LV thickness, with papillary muscles compromise in the setting of ischemic cardiomyopathy (Figure 5, Video 2). Lipid and metabolic panel were not available in our case presentation. Conservative management and optimal medical therapy including aspirin 100 mg per day, continuing with statins, angiotensin-converting enzyme (ACE) inhibitors (enalapril), and beta-blockers (bisoprolol) as maximal dose tolerated were indicated in our patient with neither angina nor dyspnea progression after two months of follow-up.

**Video 2:** Non-contrast cardiac CT findings Axial images of non-contrast CCTA showing the calcification of the LV and papillary muscle (red arrows) in the setting of prior myocardial infarction LV: left ventricle; RV: right ventricle; Ao: aorta, Cs: coronary sinus; LA: left atrium; CCTA: cardiac computed tomography angiography

## Discussion

The etiology of vascular disease in patients with HIV is multifactorial, including an increased prevalence of traditional coronary disease risk factors, dyslipidemia associated with certain antiretrovirals, insulin resistance, and endothelial dysfunction. The role of chronic inflammation and immune activation may be central to the increased risk of coronary artery disease [7]. The endothelium is a reservoir for HIV and produces cytokines such as tumor necrosis factor, interleukin-6, and free radicals in response to inflammation that in turn causes myocardial dysfunction [8]. Myocardial ischemia has been found to be an important cause of clinical or subclinical cardiomyopathy in patients with HIV infection [8,9]. Chronic inflammation plays a role, and immune activation may be central to the increased risk of coronary artery disease.

Dystrophic calcification represents the sequelae of local tissue damage and cellular necrosis. It is not associated with abnormalities in serum calcium levels or calcium homeostasis. However, hypercalcemia will aggravate the process [10]. Myocardial calcifications are uncommonly encountered by the cardiac imager and may have a range of imaging appearances, from focal calcific deposits to diffuse myocardial involvement [4]. Multimodality cardiac imaging is important to determine its presence using chest X-Ray, CT, and echocardiography. When myocardial calcifications are present, they are often related to a nonspecific and challenging etiology. Therefore, the combination of clinical features and some specific imaging patterns may help clinicians determine the underlying cause and clinical significance of the disease. The most common causes of myocardial calcification include previous MI, endomyocardial fibrosis, infections such as tuberculosis, chronic renal failure, and cardiac tumors with calcification or hyperparathyroidism [11]. No consistent data about the prevalence of myocardial calcification in HIV patients have been published.

This case provides useful evidence of the role of multimodality cardiac imaging assessment in the setting of the progression over time of an unusual dystrophic myocardial calcification after MI. We were able to demonstrate a non-ischemic involvement of the LV at the basal inferoseptal level in the context of combined cardiomyopathy in a patient with chronic HIV infection. Additionally, the stress perfusion CMR exam demonstrated the presence of inducible myocardial ischemia in the LAD territory without having a significant obstruction compatible with microvascular ischemia associated with a multivessel non-significative obstructive coronary artery disease. When assessing LV involvement, some technical characteristics of cardiac multimodality imaging can be relevant to analyze. An adequate spatial resolution and the ability of tissue characterization can improve our determination. Echocardiography is a technique with adequate spatial and temporal resolution but contrast resolution is low to moderate and it cannot assess tissue characterization precisely. In CMR imaging, the spatial resolution is about 1-2 mm, and it is 0.5-0.62 mm in CCTA. Therefore, CCTA has the best spatial resolution that enables the study of small components compared to CMR. Additionally, it is the best method for characterizing calcification in several structures, which is not possible with CMR [12].

## Conclusions

In an HIV-infected patient, cardiac and vascular coronary artery disease could be present and its diagnosis is challenging due to several associated etiologies. The dystrophic myocardial calcification can represent an unusual progression of prior MI and can be fully characterized by cardiac multimodality imaging. Echocardiography is always the first-line modality to characterize myocardial involvement and cardiac function. LGE by CMR allows the determination of the etiology of cardiomyopathy and the presence of myocardial ischemia by induced pharmacological stress protocols. CCTA is useful for the study of epicardial coronary artery disease. It is the best method to determine the degree of myocardial calcification on a thinned LV wall thanks to its high spatial resolution.
